# Neuroprotective Properties of Resveratrol and Its Derivatives—Influence on Potential Mechanisms Leading to the Development of Alzheimer’s Disease

**DOI:** 10.3390/ijms21082749

**Published:** 2020-04-15

**Authors:** Michał Wiciński, Anna Domanowska, Eryk Wódkiewicz, Bartosz Malinowski

**Affiliations:** Department of Pharmacology and Therapeutics, Faculty of Medicine, Collegium Medicum in Bydgoszcz, Nicolaus Copernicus University, M. Curie 9, 85-090 Bydgoszcz, Poland; wicinski4@wp.pl (M.W.); a.domanowska@o2.pl (A.D.); eryk.wodkiewicz09@gmail.com (E.W.)

**Keywords:** resveratrol, Gnetin C, Alzheimer’s disease, neuroprotection

## Abstract

The lack of effective Alzheimer’s disease treatment is becoming a challenge for researchers and prompts numerous attempts to search for and develop better therapeutic solutions. Compounds that affect several routes of the neurodegeneration cascade leading to the development of disease are of particular interest. An example of such substances is resveratrol and its synthetic and natural derivatives, which have gained popularity in recent years and show promise as a possible new therapeutic option in the approach to Alzheimer’s disease treatment. In this article, the state of the art evidence on the role of resveratrol (RSV) in neuroprotection is presented; research results are summarized and the importance of resveratrol and its derivatives in the treatment of Alzheimer’s disease are underlined. It also focuses on various modifications of the resveratrol molecule that should be taken into account in the design of future research on drugs against Alzheimer’s disease.

## 1. Introduction

Alzheimer’s disease (AD) became more common in an aging society. In the United States, it is estimated that the number of Alzheimer’s disease patients in 2050 will be almost 14 million [1] and over 100 million in the world [2]. Acetylcholinesterase inhibitors (rivastigmine, galantamine, donepezil) and N-methyl-D-aspartate (NMDA) receptor antagonist (memantine) are the only drugs approved by the Food and Drug Administration (FDA) for Alzheimer’s disease treatment so far. The effect of the inhibitors is based on the growth of acetylcholine in the central nervous system (CNS), while in the memantine, by blocking NMDA receptors, neurons are protected from excessive stimulation that could damage them [3]. However, it does not prevent neurodegenerative processes leading to accelerated atrophy of nerve cells responsible for the symptoms of the disease [4]. The dominant feature of the clinical picture of people affected by Alzheimer’s disease is early memory loss, which is accompanied by cognitive and behavioral disorders as the disease progresses [5]. The lack of effective Alzheimer’s disease treatment is becoming a challenge for researchers and has prompted numerous attempts to develop better therapeutic solutions in this area. There is great interest in drugs that affect not just one but several routes of the neurodegeneration cascade [6].

## 2. Aβ Peptides and Tau Protein

Histologically, Alzheimer’s disease is characterized by the presence of extracellular amyloid plaques and intracellular tangles of neurofibrillary plexuses (NFT) in certain specific areas of the brain, such as the hippocampus and association cortex of the frontal, parietal and temporal lobes [7,8]. The exact etiology of Alzheimer’s disease is not fully understood but more and more research suggests that it is a multifactorial disease [6] in which toxic Aβ aggregation [9], neuroinflammation [10], tau protein hyperphosphorylation and oxidative stress play a key role [11]. It is assumed that the key neurotoxicity inducers are not, as previously thought, large, insoluble deposits of amyloid and NFT, but soluble, nonfibrillar intermediate oligomeric forms of Aβ peptide and tau protein, which in the process of aggregation lead to disorders at the synaptic level and progressive and accelerated apoptosis of neurons [12,13]. Interestingly, researchers found that tau oligomers have the ability to enter cells and inoculate pathological aggregation of intracellular tau. What’s more, it is possible to transfer them between cells in a prion-like mechanism. This creates a chance for further induction of false folding in healthy neurons and the spread of pathology in the brain. Amyloid oligomers have similar properties, they can inoculate and stimulate monomer aggregation and lead to the formation of neurotoxic oligomers. In addition, they have a special effect on the conversion of monomeric tau to toxic tau oligomer, which initiates the cascade of events in Alzheimer’s disease [11,12,13].

Aβ peptides are produced from amyloid precursor protein (APP), which is an integral type I membrane protein, by a proteolytic route in the so-called amyloidogenic pathway using β-secretase 1 (BACE 1) and γ-secretase [14]. Recent studies have reported that the blocking of BACE1 activity may prevent the progression of cerebral amyloid angiopathy associated with abnormalities in small brain vessels caused by Aβ aggregation [15]. Resveratrol (3,5,4′-trihydroxy-trans-stilbene), a polyphenol contained in red wine, peanuts, and some berries, is known for its anti-atherosclerotic, anti-inflammatory, antioxidant, and longevity-promoting properties [16]. There is ample evidence of its neuroprotective effects (Table 1).

Resveratrol has been shown to be particularly useful for the treatment of disorders such as Alzheimer’s disease [16] because it prevents hippocampal neurodegeneration [29] and cognitive deficits [17]. Choi et al. investigated the effect of resveratrol and its oligomers isolated from Paeonia lactiflora seed on beta-site APP-cleaving enzyme 1 (BACE-1) activity in vitro. β-secretase is an enzyme of the amyloidogenic pathway, it cuts the amyloid precursor protein (APP) and thus provides the brain with toxic β-amyloid. All compounds isolated from *Paeonia lactiflora* seeds, including resveratrol (IC_50_ = 11.9 μM) proved to be effective inhibitors of β-secretase in vitro. Resveratrol trimers, gnetin H (IC_50_ = 0.34 μM) and suffruticosol B (IC_50_ = 0.88 μM), were distinguished by particularly high β-secretase inhibiting activity [30]. In turn, other researchers assessed the effect of resveratrol at a concentration of 10–40μM on the metabolism of APP in mouse neuroblastoma N2a cells expressing wild type or Swedish APP_695_. The presence of resveratrol did not change the level of APP and its C-terminal fragments C99, C89, and C83. Moreover, in cell-free tests in vitro and in culture, resveratrol did not inhibit the formation of β-amyloid. This suggests that resveratrol may not prevent Aβ formation because it does not affect β and γ-secretase activity [31]. Porquet et al. in their research used the mouse familial AD model AβPP/PS1 (amyloid-β protein precursor/presenilin 1). Resveratrol at a dose of 16 mg/kg/day was administered to AβPP/PS1 mice for 10 months, resulting in improved short-term memory in the object recognition test and a significant increase in the presynaptic protein synaptophysin, which may be an expression of improved synaptic activity. Furthermore, a significant increase in mitochondrial IV complex protein has been observed in the brain of the AβPP/PS1 mouse, which reflects mitochondrial function and constitutes neuroprotection. It is also worth noting that resveratrol treatment led to a decrease in β-secretase concentration (*p* < 0.05), without affecting AβPP, C99, and C83 [25]. Recent reports indicate that treatment with resveratrol significantly reduces the level of amyloidogenic β-secretase in mouse strains, including 3xTg-AD and non-transgenic NoTg. In addition, resveratrol contributed to an increase in the activity of the neprilysin enzyme responsible for the degradation of Aβ and promoted the increase of AMP-activated protein kinase (AMPK), peroxisome proliferator-activated receptor γ coactivator-α (PGC-1α) and phosphorylated cAMP response-element binding protein (p-CREB) in both mouse strains, which proves its neuroprotective properties [27].

Feng et al. suggest that the presence of hydroxyl groups in the resveratrol molecule and the hydrophobic interaction between resveratrol and Aβ42 may block the formation of Aβ42 fibers, but not oligomerization. Nevertheless, the authors postulate that resveratrol may have a beneficial effect on the conformation of Aβ42 oligomers and weaken their cytotoxicity. In the presence of resveratrol, the survival of SY5Y neuroblastoma cells exposed to Aβ42 oligomers was significantly higher. This effect is seen in the possibility of the direct binding of resveratrol to Aβ42 and the formation of oligomers with lower toxicity [32]. Li et al. noted the relationship between Aβ oligomers and cellular prion protein (PrPC) in disrupting the synaptic plasticity of the hippocampus. Studies in AD mice and brain tissue have confirmed the ability of soluble Aβ oligomers to bind to cellular prion protein. In contrast, the use of anti-PrPC antibodies did not impair LTP (long-term synaptic enhancement) in the presence of soluble Aβ oligomers. This suggests the involvement of PrPC in synaptotoxicity associated with Aβ oligomers [33]. Sengupta et al. in their work emphasize that Aβ oligomers act as seeds for various proteins, including PrPC, leading to the formation of toxic aggregates. Normal prion protein (PrPC) is located on the surface of the cell membrane, mainly brain neurons. In the process of incorrect folding of the cellular prion protein (PrPC), an infectious prion protein called scrapie (PrPsc) is formed, which can travel between cells and convert PrPC to PrP_SC_. The pathological PrP_SC_ prion protein has a β-sheet structure, and its important feature is its ability to aggregate. Amyloid β, α-synuclein and tau show similarity in structure and properties to prions and the propagation of incorrect folding of proteins can occur through similar mechanisms leading to the degeneration of the neural network [34].

The non-amyloidogenic route of amyloid precursor protein (APP) processing by α-secretase is an alternative to the amyloidogenic route; the activity of α-secretase results in soluble APPa product (APPsα), which is assigned neuroprotective properties [35]. The promotion of α-secretase activity seems to be beneficial in the prevention and maybe even treatment of Alzheimer’s disease, as it may counteract the formation of neurotoxic Aβ [36]. A special role in the protection of neurons against apoptosis is attributed to the nicotinamide adenine dinucleotide NAD(+)-dependent histone deacetylase SIRT1 (Sirtuin-1) [37], which regulates the function of many important transcription factors such as p53, NF-κB (nuclear factor κ-light-chain enhancer of activated B cells), and FOXO (forehead box protein O) [38,39]. Moreover, SIRT1 has been shown to reduce the deposition of Aβ due to the activation of APP processing by non-amyloidogenic pathways [40]. One of the SIRT1 activators is resveratrol [41]. A trend towards increased serum SIRT concentration was recorded in a four-week study giving rats 10 mg/kg/day of resveratrol per day (*p* = 0.011 compared to placebo) [42]. Resveratrol through SIRT-1 may lead to reduced Aβ42-induced neuroinflammation due to inhibition of the NF-κB [43] signal pathway and removal of free radicals [44].

Reduced SIRT-1 levels in the brains of Alzheimer’s disease patients are associated with the accumulation of Aβ and tau [45]. Tau is a microtubule-bound protein that provides normal neuronal function, but if it is not properly dephosphorylated it polymerizes into helical fibers forming neurofibrillary plexuses (NFT) [46]. It has been demonstrated that tau acetylation provides protection against degradation of hyperphosphorylated tau and the presence of SIRT 1 is an important factor leading to in vitro and in vivo tau deacetylation [47]. Resveratrol via SIRT-1 can, therefore, be expected to reduce the level of hyperphosphorylated tau and provide protection against neurodegeneration. In addition, it has been noted that resveratrol by lowering the expression of MID1 ubiquitin ligase increases protein phosphatase 2A (PP2A) activity and promotes tau dephosphorylation by preventing its accumulation [48].

## 3. Oxidative-Nitrosative Stress

It is believed that the first factor preceding the appearance of pathological changes characteristic for Alzheimer’s disease is oxidative damage to cellular structures [49]. Oxidative stress, and especially lipid peroxidation, seems to be molecular pathways involved in the early stages of the disease [50]. It has been found out that oxidative stress induces BACE1 expression in a mechanism dependent on γ-secretase activity through the c-*jun* N-terminal kinase JNK/*c-jun* pathway, thereby enhancing the production of Aβ peptides; while, Aβ intensifies oxidative stress, which leads to the formation of a so-called vicious circle [51]. In the presence of oxidative stress, superoxide anions are also produced by neuronal nitric oxide synthase (nNOS), which then react with NO generating reactive nitrogen species in the form of peroxynitrites with strong oxidizing properties [52]. There is evidence that Aβ through oxidative-nitrosative stress leads to the damage of brain endothelial cells DNA (deoxyribonucleic acid) [53]. It is suggested that the improvement of spatial memory of rats with Alzheimer’s disease, treated with resveratrol, is the result of its ability to effectively reduce the level of oxidative stress and protect neurons from apoptosis. This study showed that resveratrol abolishes Aβ-induced lipid peroxidation and expression of heme oxygenase-1 (HO-1) reduction; these effects are attributed to resveratrol’s ability to reduce inducible nitric oxide synthase (iNOS) levels in the hippocampus [18].

**Figure 1:**
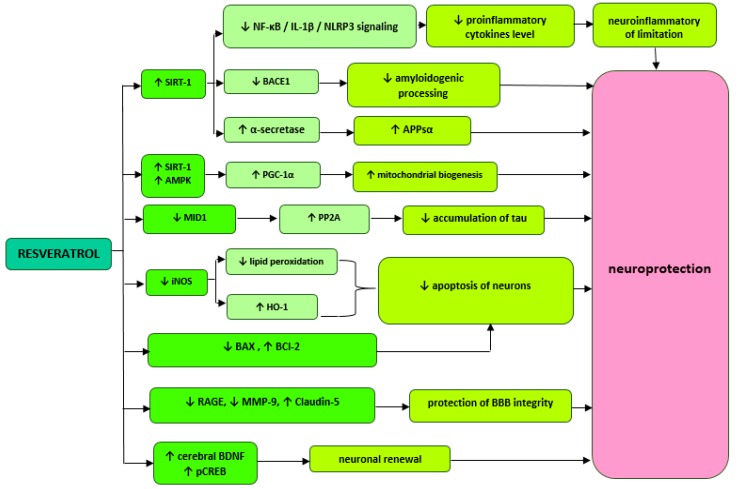
Proposed mechanisms of resveratrol activity. ↓—reduction,↑—increase, SIRT1 (Sirtuin-1), NF-**κ**B—nuclear factor kappa-light-chain-enhancer of activated B cells, BACE 1—beta-site APP cleaving enzyme 1, AMPK—AMP-activated protein kinase, PGC-1—peroxisome proliferator-activated receptor γ coactivator-α, MID1—ubiquitin ligase, iNOS—inducible nitric oxide synthase, HO-1—heme oxygenase-1, BCl-2—B-cell lymphoma 2, BAX—BCL2-associated X protein, PDE—phosphodiesterase 4, RAGE—glycation end products, MMP-9—metalloproteinase 9, BDNF—brain-derived neurotrophic factor.

Wang et al. report that improved memory performance in AD mice treated with resveratrol may be associated with the inhibition of phosphodiesterase 4 (PDE4) subtypes A5, 4B1, and 4D3 expression and a subsequent increase in activation of the cAMP/PKA (protein kinase A) pathway. Furthermore, increased cAMP (cyclic adenosine monophosphate) activity may mediate in the reduction of neuronal neuroinflammation and apoptosis. After three weeks of administration of 40 mg/kg resveratrol to AD mice, statistically significant reductions in the levels of pro-inflammatory cytokines IL-1β and IL-6, an increase in BCl-2 antiapoptotic protein expression, and a decrease in Bax (BCL2-associated X protein) proapoptotic protein expression were achieved. In addition, resveratrol has been shown to eliminate the negative effects of Aβ42 on phosphorylated cAMP binding protein (pCREB) and brain-derived neurotrophic factor (BDNF), which ultimately results in memory enhancement in mice and speaks for the neuroprotective effect of resveratrol [19]. This polyphenol, administered orally to healthy rats at a dose of 10 mg/kg for four weeks, also increases BNDF levels. The study showed a significant increase in BNDF concentration (1.64 ± 0.31 ng/mL, *p* = 0.031) in the serum compared to the control group (1.32 ± 0.26 ng/mL). In addition, it has been shown that resveratrol can directly reduce contractility of smooth muscle cells in rat tail arteries in a manner independent of endothelial NO synthase (NOS-3). Thanks to such properties, resveratrol can have positive effects on maintaining stable cerebral blood flow and thus prevent neuronal damage and cognitive function deficits [24]. Resveratrol administered in the cerebral ventricle at a dose of 0.02 mg/kg/day improves memory and reverses Aβ-induced changes in the inflammatory response and mitochondrial dysfunction. Researchers achieved a significant reduction in the levels of NF-κB (nuclear factor κ-light-chain enhancer of activated B cell), interleukin 1β and NLRP3 (NOD-, LRR- and pyrin domain-containing protein 3) inflammation markers in the hippocampus and cortex of mouse with AD. AMP-activated protein kinase (AMPK) and peroxisome proliferator-activated receptor γ coactivator-α (PGC-1α) activities that are involved in mitochondrial biogenesis have also increased [26]. The simplified diagram with possible mechanisms of resveratrol is presented in Figure 1.

## 4. Central Nervous System Inflammation and Integrity Disorders of the Blood–Brain Barrier (BBB)

Recently, much attention has been focused on brain endothelial cell dysfunction and microcirculation disorders present in the early stages of Alzheimer’s disease [54]. It has even been hypothesized that it is vascular pathology that precedes dysfunction and degeneration of neurons [54]. A properly functioning endothelium determines the integrity of the blood–brain barrier (BBB) and maintains neurovascular balance [55]. It is known that Aβ acts on the endothelium, which results in the remodeling of smooth muscle cells of small cerebral arterioles and their excessive contractility [56] leading to autoregulation disorders, a decrease in cerebral blood flow (CBF), and ultimately to cognitive impairment and dementia [57]. In fact, AB’s ability to cause changes in vascular structure and destroy BBB integrity is associated with oxidative stress [58] through their association with reactive oxygen species (ROS) producing NADPH oxidase activity [57], which has significant deposits in brain blood vessels [59]. A violation of BBB integrity disrupts Aβ transport contributing to the excessive accumulation of toxic Aβ in the brain and further aggravating pathology [60]. Matrix metalloprotein-9 (MMP-9) and tight junction protein Claudin-5 have been shown to regulate BBB permeability and play an important role in neuropathologies such as Alzheimer’s disease [61,62]. In the rat Alzheimer’s disease model, resveratrol defends BBB integrity by reducing advanced glycation end products (RAGE), matrix metalloprotein-9 (MMP-9) and increasing Claudin-5 but also reduces neuroinflammation by affecting nuclear factor NF-κB expression [63]. It has been noticed that microglia and neuroinflammation are activated around the amyloid plaques. Resveratrol at a dose of 350 mg/kg effectively prevents the activation of microglia in the brain of APP/PS1 mouse. It is suggested that this effect is achieved through the inhibitory effects of resveratrol on the TLR4 (toll-like receptor)/NF-κB/STAT (signal transducer and activator of transcription) signaling cascade [28]. Phase two of clinical tests among people with mild or moderate Alzheimer’s disease confirm resveratrol’s ability to modulate neuroinflammation and protect blood–brain barrier integrity [20]. Resveratrol restores BBB integrity by significantly reducing the level of matrix metalloprotein-9 (about 50%) in cerebrospinal fluid (CSF), which can provide adequate Aβ clearance and limit the inflow of inflammatory mediators into the central nervous system (CNS) [20]. Based on changes of Aβ40 (*p* = 0.024) in plasma and Aβ40 in CSF (*p* = 0.002) compared to placebo, resveratrol is suggested to cross the BBB and affect the CNS [64]. It was observed that high-dose resveratrol treatment was paradoxically associated with greater brain volume loss [64]; on this basis, it was hypothesized that pseudoatrophy is the result of a reduction in CNS edema due to the strong anti-inflammatory effect of resveratrol [20,21].

## 5. Metabolic Disorders

The high-fat and high-glucose diets’ effect on the progression of Alzheimer’s disease due to metabolic dysfunction is being considered [65]. Factors of cardiovascular diseases e.g., diabetes, hypertension, and lipid disorders promote the development of neurodegenerative diseases, including Alzheimer’s disease [66,67,68,69]. In turn, the Mediterranean diet plays an important role in the prevention of Alzheimer’s disease [66], among others due to its protective effect on the vascular endothelium through precious polyphenols such as resveratrol and unsaturated fatty acids [70]. A relationship between high glucose levels and lower memory performance and reduced volume of the human hippocampus [71], which is particularly susceptible to carbohydrate metabolism disorders, has been shown [72]. Experimental results may indicate a beneficial effect of resveratrol treatment on improving glycemic control among patients with type 2 diabetes mellitus [73]. After three months of regular resveratrol supplementation (250 mg/d), a statistically significant reduction in HbA1c (*p* < 0.05), systolic blood pressure and total cholesterol levels [73], i.e., risk factors for Alzheimer’s disease [68], were observed. There is evidence that resveratrol by regulating glucose metabolism can positively affect brain functions and protect neurons [22]. It can be assumed that this effect is associated with the activation of AMP-activated protein kinase (AMPK) and SIRT1, which are known to regulate insulin sensitivity and mitochondrial biogenesis [74]. It is noted that the metabolic effects of resveratrol depend on AMPK [75]. Resveratrol by activating AMPK increases the level of NAD^+^ by indirectly activating SIRT1 [76]. The upregulation of SIRT1 by resveratrol has been reported to prevent oxidative stress-induced endothelial cells from aging and thus may have positive effects on inhibiting atherosclerosis [77].

## 6. Resveratrol Derivatives with Possible Neuroprotective Effects

### 6.1. Resveratrol Modified with Selenium Nanoparticles

Selenium is an element necessary for the proper development and function of the brain [78]. Selenium-containing nanoparticles have been reported to be effective in treating Alzheimer’s disease [79]. Aβ toxicity and oxidative stress strongly correlate with neurodegenerative pathology and the cascade of many adverse events leading to Alzheimer’s disease development [80]. Studies have shown that elemental selenium at nano size (Nano-Se) has high antioxidant efficacy and significantly less toxicity compared to selenomethionine (SeMet) (LD_50_NanoSe 92.1 mg Se/kg and LD_50_SeMet 25.6 mg Se/kg, respectively) [81]. Furthermore, sialic acid (SA)-modified selenium nanoparticles coated with B6 peptide effectively inhibit Aβ aggregation and promote disaggregation of fibrils into non-toxic oligomers [82]. It is suggested, that it is a promising therapeutic option in the treatment of brain diseases [82]. Similar results were obtained using stabilized epigallocatechin-3-gallate selenium nanoparticles coated with Tet-1 peptide [83]. The obtained effects are attributed to the unique properties and structure of nanomaterials; electrostatic interactions and a large surface are unfavorable conditions for the growth of peptide fibrils due to the lack of contact between monomers [5]. In the case of polyoxometalates, these properties proved to be the key to effective inhibition of polymerization at the level of Aβ monomers [84]. Interestingly, the use of nanoparticles increases the stability of resveratrol in vivo, and thus its therapeutic potential [85]. It was noted that the reaction between Aβ and a redoxoactive metal ion (Cu^2+^, Zn^2+^, and Fe^2+^) enhances the aggregation of Aβ and promotes the formation of ROS [86]. Yang et al. in their study assessed the ability of resveratrol and resveratrol modified with selenium nanoparticles to inhibit Cu^2^-induced aggregation and cytotoxicity of Aβ42. Comparing the obtained results, it can be stated that the use of selenium nanoparticles significantly improved the anti-aggregation capabilities of resveratrol. The viability of PC12 cells in the presence of resveratrol and resveratrol-functional selenium nanoparticles (RSV-SeNP) was 78% and 93%, respectively, relative to the control (100%). It is believed that the more attractive ability of RSV-SeNP to inhibit the cytotoxicity induced by Aβ42–Cu^2+^ is due to its better antioxidant properties [87].

### 6.2. Resveratrol Hybrid Compounds

Multifunctional compounds, as multi-targeted ligands (MTDL), have been reported to appear to be a promising therapeutic option in neurodegenerative diseases, including Alzheimer’s disease [88]. Many hybrid compounds with different pharmacophores have been developed (Table 2) and their in vitro biological activity has been evaluated.

It is known that the deposition of Aβ in the brain stimulates astrocytes and microglia leading to neurosurgitis and neuronal apoptosis [93,94]. As previously mentioned, Cu^2+^, Zn^2+^, and Fe^2+^ dyshomeostasis accelerates Aβ aggregation and causes oxidative stress [95] associated with neuronal degeneration [89]. Therefore, a strategy focused on these molecular Alzheimer’s disease goals may be appropriate. Cheng et al. obtained by replacing the resveratrol benzene ring with a metal-maltol chelating group, a series of hybrids and assessed their ability to inhibit aggregation of Aβ42, antioxidant and metal-binding with positive effects. Compound B inhibited the aggregation of Aβ42 at IC_50_ = 8.29 μM (IC_50_ resveratrol = 11.89 μM) and promoted disaggregation of Aβ induced by Fe^3+^ ions (*p* < 0.01 relative to control) [96]. Other researchers, on the other hand, used deferiprone, an oral iron-chelating drug, among patients with thalassemia [97] to synthesize hybrids with resveratrol [90]. Hybrid C was distinguished by a high inhibition of Aβ42 aggregation induced by Cu^2+^/Fe^3+^ ions (decrease in ThT fluorescence to 57% and 64%, *p* < 0.01, for resveratrol 86% with copper and 87% with iron, respectively) [90]. What’s more, compound D hybrid pyridoxine and resveratrol, a Mannich base derivative, in addition to good antioxidant properties (ORAC: 2.56) had a dual inhibitor function; it inhibited both acetylcholinesterase (AchE) (IC_50_ = 2.11 μM) and monoamine oxidase B (MAO-B) (IC_50_ = 12.4 μM) [91]. It has been noted that MAO-B may be responsible for the development of Alzheimer’s disease [98], because one of the products of reactions catalyzed by MAO-B is hydrogen peroxide, which can become a source of reactive oxygen species [99]. Jeřábek et al. combined resveratrol with the cholinesterase inhibitor tacrine (compound E). Good hAChE inhibitory ability, anti-Aβ42 aggregation and protective effects on primary cultures of lipopolysaccharide (LPS)-treated astrocytes were obtained. Despite the many advantages of this combination, clinical use may be limited due to hepatotoxic potential [92].

### 6.3. Melinjo and Gnetin C Extracts

Gnetin C is a natural stilben, resveratrol dimer, isolated among others from seeds of the Melinjo plant (*Gnetum gnemon* L.) [100] and XinJiang vine stems [101]. Melinjo seeds are an essential ingredient in Indonesian food and contain a mixture of stilbenoids (Table 3) with excellent free radical scavenging properties as well as antimicrobial activity against enterobacteria and food microorganisms [102].

In addition, vasoprotective properties [104] and anti-tumor melinjo [105] extracts have been reported. Kunimasa et al. showed in their studies that Gnetin C can inhibit proliferation (*p* < 0.05) and tube formation (IC_50_ = 2.6 μM) of human umbilical vein endothelial cells (HUVEC) much more strongly than resveratrol (IC_50_ = 28.9 μM). It has been observed that the antiproliferative properties of Gnetin C may be due to its ability to inactivate extracellular signal-regulated kinase ERK1/2 induced by vascular endothelial growth factor and basic fibroblast growth factor (VEGF-bFGF) [106]. RAF-MEK1/2-ERK1/2 signaling disorders have been reported to promote tumor development, and ERK1/2 inhibitors are an effective weapon in the fight against this disease [107]. In their work, Syota et al. assessed the effect of treatment with 20 μM Gnetin C, ε-viniferin, and resveratrol on the production of Aβ42 monomers in human neuroblastoma cells SH-SY5Y. Gnetin C showed the best effectiveness; it reduced the production of Aβ42 monomers by as much as 63%, while ε-viniferin and resveratrol by 34% and 33%, respectively. This effect is attributed to the ability to inhibit BACE-1 expression (*p* < 0.05) and induction of expression of MMP-14 (matrix metalloproteinase-14), an Aβ degrading enzyme (*p* < 0.05) [108]. Ikuta et al. in their studies in mice compared the use of a high-fat diet with a combination of this diet and 2.0% MSE^*^. After eight weeks, the results in both groups were evaluated. In the MSE group, a significant reduction in weight gain (*p* < 0.001), blood insulin (*p* < 0.01) and HOMA-IR index (*p* < 0.05) was achieved, however, without affecting the corresponding lipoprotein fractions (Table 4). Furthermore, it was noted that supplementation with 0.2% melinjo extract containing Gnetin C in mice fed high-fat diets reduced the risk of death by 25% (*p* = 0.036) [109]. Recent studies show that high doses of MSE (5000 mg) in powder form are safe for humans and are not associated with serious adverse events [103]. In double-blind, randomized controlled study Konno et al. studied the beneficial effects of using 750 mg of MSE powder by healthy Japanese men. After eight weeks of treatment, a statistically significant decrease in uric acid level (*p* < 0.05) and an increase in serum HDL cholesterol (*p* < 0.05) were obtained, without affecting LDL cholesterol. Interestingly, it has been shown that the ability of MSE and Gnetin C to reduce uric acid may be due to the inhibition of AT1 activity, not xanthine oxidase. It is suggested that by agonistic action on peroxisome proliferator-activated receptor (PPARα and PPARγ), MSE leads to an increase in HDL levels [104]. The safety of pure Gnetin C (150 mg/day), its better bioavailability than resveratrol and the ability to lower uric acid in serum was confirmed in a randomized phase one clinical test. It is worth noting that two-week supplementation of pure Gnetin C by healthy people was associated with a significant reduction of HDL-C (*p* = 0.025), LDL-C (*p* = 0.011) and adiponectin (*p* = 0.000087) in serum compared to placebo, which is in contradiction to previous studies and appears to promote the progression of atherosclerosis [110]. Therefore, further research is needed to determine the effect of Gnetin C on the metabolic profile in humans. Other researchers evaluated the effect of using MSE in healthy individuals on plasma oxidative stress parameters. After 28 days of administration of MSE containing 262 mg Gnetin C and 5.8 mg resveratrol per day, the plasma antioxidant capacity was 2.5 times higher than at the beginning of the study (*p* = 0.01). In plasma samples taken after two weeks of MES ingestion, the average concentration of Gnetin C (0.344 ± 0.205 µg/mL) was significantly higher than that of resveratrol ((0.0612 ± 0.0535 µg/mL). In addition, Espinoza et al. examined the expression of proinflammatory cytokines from peripheral blood mononuclear cells (PBMCs) cultured with or without MES in vitro. It has been observed that MSE inhibits the secretion of interferon-γ (IFN-γ) and tumor necrosis factor α (TNF-α) from PBMCs activated with phytohemagglutinin A (PHA) in a concentration-dependent manner. The results obtained may indicate good in vivo antioxidant and in-vitro anti-inflammatory properties [111].

Nakagami et al. observed that the plasma concentration of Gnetin C on the 14th day of supplementation (150 mg/day) was in the range of 601 to 2490 ng/mL, and 14 days after the last dose in the range of 27–98 ng/mL, and its concentration monoglucuronide metabolite in the range 86–862 ng/mL [110]. However, in the case of resveratrol Walle et al. reported that it undergoes intensive and rapid metabolism in the human body, which means that its bioavailability is very low, and only a trace amount of unchanged resveratrol (<5 ng/mL) and quite high concentration of the metabolites 491 +/− 90 ng/mL (about 2 μM) remain in plasma after oral administration of 25 mg. Maximum total plasma radioactivity was 491 +/− 90 ng/mL and was reached 1 h after oral administration. Interestingly, resveratrol absorption from the gastrointestinal tract is large and amounts to at least 70% [112]. Other researchers, in turn, administered 50 mg of resveratrol to rats to assess its tissue distribution. After 2 h, plasma resveratrol concentration accounted for 1.7% of the total dose administered, and after 18 h only 0.5%. The highest resveratrol concentration 2 h after administration was found in the liver (1%) and kidneys (0.6%), while in the brain less than 0.1% (about 0.03%) of the dose administered was found, with resveratrol concentration in brain tissue showing a slower decrease compared to liver and kidneys [113].

## 7. Conclusions

The information presented in this article allows resveratrol and its derivatives to be considered as potential neuroprotective agents capable of affecting many pathological cascade targets leading to the development of Alzheimer’s disease. Inhibition of the aggregation of toxic Aβ appears to play a key role in reducing AD-related neurodegeneration. Free radical scavenging and anti-inflammatory properties may be helpful in preventing cerebrovascular endothelial dysfunction and maintaining normal blood–brain barrier integrity. When designing new drugs against Alzheimer’s disease, it is worth considering the use of modifications based on selenium nanoparticles and hybrid resveratrol derivatives with its beneficial effects. The modified molecules showed better antioxidant and anti-aggregation activity in vitro. If their potential effect on the protection of neuronal cells is proven in vivo research, they may find use in the treatment of brain diseases. The fact that resveratrol is extensively metabolized in the body and its bioavailability is low, significantly limits its clinical use. Gnetin C, a natural stillben, resveratrol dimer contained in the seeds of *Gnetum gnemon* L. has better pharmacokinetic properties. If the ability of pure Gnetin C to prevent cardiovascular diseases associated with metabolic syndrome was proven, this compound could prove useful in reducing the burden of certain neurodegenerative diseases, including Alzheimer’s disease. Additional studies are necessary to verify the effect of Gnetin C on the metabolic profile in humans. However, for any incurable disease medicine to be effective, it is necessary to know and understand its exact mechanism, which in the case of Alzheimer’s disease is still unclear.

## Figures and Tables

**Table 1:** Neuroprotective properties of resveratrol in animal and human studies.

Subject of the Study	Dose	Effect	Reference
Streptozotocin-induced Alzheimer’s dementia model in Wistar rats	10 i 20 mg/kg of resveratrol per day i.p.	↑glutathione in brain	[17]
Sprague–Dawley rats with AD	100 µM i.c.v.	↑HO-1 ↓iNOS in hippocampus	[18]
ICR mice with AD	40 mg/kg of resveratrol per day	↓PDE4A5,4B1,4D3 expression ↑BDNF ↑pCREB ↑PKA ↑BCl-2 expression ↓BAX expression ↓IL-1β, IL-6 in hippocampus	[19]
People with mild or moderate AD	max 2 g/day of resveratrol	↓Aβ40 in plasma and in cerebrospinal fluid resveratrol safe and well tolerated	[20]
People with mild or moderate AD	max 2 g/day of resveratrol	↓MMP-9 in cerebrospinal fluid	[21]
Healthy overweight elderly man (BMI 25–30 kg/m^2^)	200 mg/day of resveratrol 320 mg of quercetin	↑memory performance ↑functional connectivity (FC) of the hippocampus ↓HbA1c in serum	[22]
People with mild or moderate AD	5 mg resveratrol 5 g malate 5 g dextrose/twice a day	resveratrol safe and well tolerated	[23]
Wistar rats	10 mg/kg of resveratrol per day	↑serum BDNF	[24]
AβPP/PS1 mouse model of AD	16 mg/kg of resveratrol per day	↑synaptophysin ↑mitochondrial IV complex protein in brain	[25]
Mouse model of AD induced by Aβ1–42	0.02 mg/kg of resveratrol per day for cerebral ventricle	↑AMPK ↑PGC-1 ↓NF-κB / IL-1β / NLRP3 in hippocampus and prefrontal cortex	[26]
Triple-transgenic mouse model of AD (3 x Tg-AD)	100 mg/kg of resveratrol per day	↑neprilisine ↓BACE1 ↑AMPK ↑PGC-1 ↑pCREB in hippocampus	[27]
APP/PS1 mouse model of AD	350 mg/kg resveratrol once a day	inhibition of microglia activation by Aβ in brain	[28]

↓—reduction,↑—increase, i.p.—intraperitoneal injection, i.c.v—intracerebroventricular injection, HO-1—heme oxygenase-1, iNOS—inducible nitric oxide synthase, BDNF—brain-derived neurotrophic factor, AβPP/PS1—amyloid-β protein precursor/presenilin 1, pCREB—phosphorylated cAMP response-element binding protein, BCl-2—B-cell lymphoma 2, BAX—BCL2-associated X protein, IL-1β—cytokine interleukin-1β, IL-6—interleukin 6, MMP-9—metalloproteinase 9, HbA1c—glycated hemoglobin A1c, AMPK—AMP-activated protein kinase, PGC-1—peroxisome proliferator-activated receptor γ coactivator-α, NF-κB—nuclear factor κ-light-chain enhancer of activated B cell, NLRP3—NOD-, LRR- and pyrin domain-containing protein 3, BACE1—β-secretase 1, APP—amyloid precursor protein, PKA—protein kinase A, PDE 4—phosphodiesterase 4.

**Table 2:** Resveratrol hybrid compounds in studies in vitro.

Compound Symbol	Resveratrol Hybrid	References
**B**	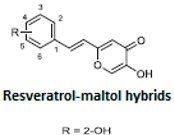	[89]
**C**	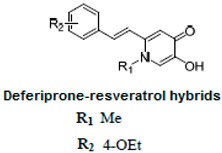	[90]
**D**	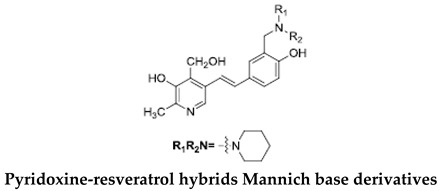	[91]
**E**	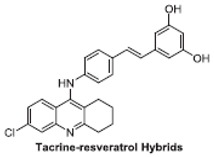	[92]

ThT—thioflavin-T based fluorometric assay, ORAC—oxygen radical absorbance capacity.

**Table 3:** Resveratrol derivatives in MSE [103] and chemical structure of resveratrol dimer derivatives [102].

Resveratrol Derivatives in MSE	Chemical Structure of Resveratrol Dimer Derivatives
Trans-resveratrol Trans-piceid Isorhapontigenin	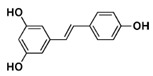 **resveratrol**
**Resveratrol Dimers**: **GC**: Gnetin C **GL:** Gnetin L **GMA:** Gnemonoside A **GMC:** Gnemonoside C **GMD:** Gnemonoside D	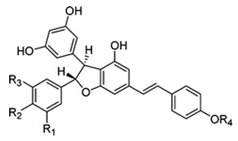 **GC**: R_1_ = R_3_ = R_4_ = H, R_2_ = OH **GL**: R_1_ = OCH_3_, R_2_ = R_4_ = H, R_3_ = OH **GMA**: R_1_ = R_3_ = H, R_2_ = OGlc, R_4_ = Glc **GMC**: R_1_ = R_3_ = R_4_ = H, R_2_ = OGlc **GMD**: R_1_ = R_3_ = H, R_2_ = OH, R_4_ = Glc

**Table 4:** MSE and Gnetin C in vivo studies.

Subject of Study	Dose (p.o)	Duration of Treatment	Effect	Reference
Healthy man	750 mg/day of MSE powder (>20% resveratrol derivatives, including only 0.75 mg/day of resveratrol)	8 weeks	↑HDL ↓uric acid in serum	[104]
Diet-induced obesity mouse model	High-fat diet (HFD) + 2.0% MSE	8 weeks	↓weight gain ↓insulin in blood ↓HOMA-IR	[109]
Healthy person	150 mg/day of Gnetin C	2 weeks	↓uric acid ↓HDL ↓LDL ↓adiponectin in serum well tolerated	[110]
Healthy person	320 mg/day of MSE (262 mg (2.57%) of Gnetin C 5.8 mg (0.09%) of trans-resveratrol)	28 days	↑serum antioxidant activity well tolerated	[111]

MSE—*Melinjo* seed extracts (resveratrol dimers: gnemonoside A, C, D; gnetin C; trans-resveratrol; other resveratrol derivatives), HDL—high density lipoprotein, LDL—low density lipoprotein, HOMA-IR—homeostasis model assessment of insulin resistance.
